# Improvements for the solution of crack evolution using extended finite element method

**DOI:** 10.1038/s41598-024-76626-0

**Published:** 2024-11-06

**Authors:** Yuxiao Wang, Akbar A. Javadi, Corrado Fidelibus, Huiqi Liang

**Affiliations:** 1 Department of Engineering, University of Exeter, Harrison Building, North Park Road, Exeter, EX4 4QF United Kingdom; 2 Department of Innovation Engineering, University of Salento, Complesso Ecotekne, Strada per Monteroni, Lecce, 73100 Italy; 3 School of Civil Engineering, Southeast University, 2 Southeast University Road, Nanjing, 211189 China

**Keywords:** Extended finite element method, Crack evolution, Symmetric nodes, Accuracy improvement for the interaction integral method, Applied mathematics, Civil engineering

## Abstract

It is demonstrated that the eXtended Finite Element Method (XFEM) is of remarkable efficiency in simulating crack evolution by eliminating the need for remeshing and refinement. In this paper, it is shown how to enhance the solution efficiency through a comprehensive mathematical investigation of the solution process using XFEM. A typical example is presented to illustrate the disparities in nodal displacements along the two symmetric faces of the crack resulting from the approximation of XFEM. By analysing the structure and components of the global stiffness matrix, the underlying causes of these discrepancies are identified. Building upon these findings, two improvements of the solution are proposed to gain an acceptable accuracy in computing the nodal displacements. The first improvement consists of the subdivision of the enriched elements depending on the characteristic of the distribution of Gauss points. The second improvement is set by determining the optimal number of Gauss points in each sub-element near the crack tip. To calculate the stress intensity factor of the crack under surface pressure, such improvements are applied in conjunction with the interaction integral method, which significantly reduces computational time and eliminates the influence of surface tractions. The numerical solution is validated by comparing it with the analytical solution and the standard XFEM solution. The proposed improvements can enhance both the accuracy of the solution and the computational efficiency of XFEM.

## Introduction

In recent decades, diverse numerical solutions have been proposed for the numerical simulation of crack propagation and evolution in the framework of Fracture Mechanics (FM) engineeristic problems. Apart from the application of more conventional methods, such as Boundary Element Method^1,2^, Discrete Element Method (DEM)^3^, Element Free Galerkin Method (EFGM)^4^, and Finite Element Method (FEM)^5,6^, in more recent years, more advanced methods have been developed, including the Phase Field Method (PFM), the Smoothed Finite Element Method (S-FEM), the Cracked Particle Method (CPM) and the eXtended Finite Element Method (XFEM)^7,8^.

In PFM^9–11^, the crack propagation is simulated by using a continuous scalar field, named the phase field, for a smooth transition from the intact material (phase field equal 1) to a fully broken material (phase field equal 0).With PFM an explicit crack tracking is not necessary and crack branching and merging are seamlessly reproduced. It is highly versatile, applicable to brittle, quasi-brittle, and ductile materials, and extendable to handle dynamic loading, large deformations, and various boundary conditions. However, by introducing the phase field as additional variable, the size of the equation system increases and a fine mesh near the cracks is usually required. S-FEM^12,13^ is an advanced FEM characterized by the incorporation of a smoothing operation over the strain field, that is computed by employing strain smoothing over element subdomains, rather than with shape functions. With S-FEM, the stress concentrations near the crack tips are better computed for a more adequate prediction of crack initiation and propagation.However, for the smoothing operations additional computational steps are introduced, thus the computational demand increases at least for large-scale problems. The formulation and implementation of S-FEM are rather complex, requiring a deep understanding of the smoothing techniques and the underlying mathematical principles. The performance depends on the choice of smoothing parameters and techniques of smoothing (e.g., node-based, edge-based, or cell-based smoothing). A relatively recent numerical method introduced for fracture mechanics is CPM^14,15^, belonging to a class of particle-based methods for which a crack is modeled through the interaction of particles rather than relying on continuous fields or explicit crack tracking. This method is particularly effective in capturing complex crack patterns, such as branching, merging, and fragmentation. There is no need for crack tracking or remeshing, complex fracture patterns, such as branching, merging, and crack coalescence are easily accommodated without requiring pre-defined crack paths. However, for even simple problems for the application a fine particle discretization is required to accurately capture the crack growth, leading to significant computational costs, especially for large-scale problems. Additionally, the interactions between many particles increase the complexity and runtime of simulations.

Compared to all the above-mentioned methods, XFEM offers great advantages; it is built upon the principles of the Partition of Unity Method (PUM)^16–18^ which involves introducing enrichment functions into standard elements to capture the material discontinuity accurately. The displacement approximation comprises a continuous component and a discontinuous component. A standard FEM representation is employed to calculate the continuous component, while additional enriched functions are used for the discontinuous component.

XFEM originated from the pioneering work of Belytschko and Black^19^. Later, Moës et al^20^ introduced the step functions as an enrichment method to represent the crack discontinuity and employed the Westergaard function to account for the tip features. Daux et al^21^ proposed a new discontinuous function that incorporates enrichment approximation to address multiple branched cracks and intersecting cracks. In XFEM, the need for remeshing and mesh refinement is circumvented, thus substantial savings are gained in computational costs and time for numerical simulation.

To enhance the accuracy of XFEM approximation, researchers have explored various optimization strategies. Song et al^22^ modified the basis of XFEM by introducing phantom nodes, superposed elements, and nodal degrees of freedom to effectively represent the crack discontinuity. Moës et al^23^ proposed a modified level set method, using the absolute value of the level set as the enrichment parameter. Liu et al^24^ employed multiple layers of enriched nodes around crack tips to reduce global errors. Fries^25^ proposed a modified enrichment function applicable to specific element types and partial differential equations.

In this paper, first, by adopting the enrichment function developed by Moës et al^20^, an investigation is reported about the nodal displacements on both sides of a central crack subjected to surface stress, concerning the asymmetrical characteristics of such displacements. Following the investigation, a new strategy is proposed to ensure the symmetry of the nodal displacements and enhance the calculation accuracy. The proposed strategy consists of 1) the characteristic of the distribution of Gauss points within the element (such as symmetry and proportionality) is used to assemble the stiffness matrix for the purpose of saving extra computation costs^26,27^, and 2) conversion of the line integral for a crack subjected to surface stress into an area integral by using known enriched elements as calculating such a line integral is quite challenging and detailed calculation procedures are lacking in the literature^28,29^. In addition, different numbers of Gauss points are tested to find the most reasonable number for the final result. In this respect, previous contributions focused on the modification of the enrichment functions or the employment of different enrichment methods, whereas the strategy proposed herein has a different target and as such constitutes a novel contribution.

The paper is structured as follows: in Section 2, the basis of XFEM is illustrated; in Section 3, the solution for the nodal displacements in a central-crack model is investigated; in Section 4 the sources of the asymmetry of the solution are identified and the improvements of the solution are described; a few statements concerning the convergence of XFEM with the proposed improvements are also given; in Section 5, the numerical results in terms of Stress Intensity Factor (SIF) around a central-crack tip obtained by applying the proposed improvements are compared with the analytical solution and the standard XFEM solution, and, in addition, the simulated crack propagation is reported; finally, in Section 6, concluding remarks are furnished.

All the simulations described herein were performed with a dedicated code developed by the first author^30,31^.

A glossary of terms used herein is reported in Table 1.

**Table 1:** Glossary of terms.

Term	Symbol
displacements of the $n^\textrm{H}$ nodes	$\textbf{a}$
domain of integration for the interaction integral, first part	*A*
domain of integration for the interaction integral, second part	$A_1$
constant relative to a Gauss point in Equation 11	$A_\textrm{Gp}$
displacements of the $n^\textrm{tip1}$ and $n^\textrm{tip2}$ nodes	$\textbf{b}^1$, $\textbf{b}^2$
strain differential operator matrix	$\textbf{B}$
component of $\textbf{B}$ relative to the Heaviside-enriched nodes	$\textbf{B}_\textrm{a}$
component of $\textbf{B}$ relative to the tip-enriched nodes	$\textbf{B}_\textrm{b}$
component of $\textbf{B}$ relative to the standard nodes	$\textbf{B}_\textrm{u}$
coefficients in Equation 13	$c_i$,$P_i(1)$,$P_i(2)$
elasticity tensor	$\textbf{D}$
single Heaviside element at one extremity of the crack	e$^\textrm{H}$
single crack-tip element at one extremity of the crack	e$^\textrm{tip}$
elastic modulus	*E*
relative error in energy norm	$E_\textrm{er}$
level set function	$f(\textbf{x})$
body forces, surface forces on $\Gamma$, concentrated forces	$\textbf{F}_\textrm{b}$, $\textbf{F}_\textrm{s}$, $\textbf{F}$
Heaviside enrichment function	$H(\textbf{x})$
interaction integral	$I^{(1,2)}$
Heaviside-enriched elements and tip-enriched elements contribute to $I^{(1,2)}$	$I^\textrm{H}$,$I^\textrm{tip}$
global stiffness matrix	$\textbf{K}$
effective, Mode-I, and Mode-II Stress Intensity Factors (SIFs)	$K_\textrm{eff}$, $K_\textrm{I}$,$K_\textrm{II}$
crack toughness Mode-I	$K_\textrm{IC}$
length of the central crack	*l*
differential operator	$\textbf{L}$
normals	$\textbf{m},\textbf{n}$
number of standard nodes	*n*
standard shape functions	$N(\textbf{x})$
number of Heaviside enriched nodes	$n^\textrm{H}$
number of Heaviside-enriched elements	$n^\textrm{H}_\textrm{e}$
number of tip-enriched nodes	$n^\textrm{tip1}$,$n^\textrm{tip2}$
number of tip-enriched elements	$n^\textrm{tip}_\textrm{e}$
load vector	$\textbf{P}$
weighting function	*q*
polar coordinates from a crack tip	*r*,$\theta$
radius of a small circle surrounding a crack tip	$r_\textrm{s}$
displacements	$\textbf{u}(\textbf{x})$
current displacements, strains, and stresses	$\textbf{u}^{(1)}$,$\varvec{\upvarepsilon }^{(1)}$,$\varvec{\upsigma }^{(1)}$
auxiliary displacements, strains, and stresses	$\textbf{u}^{(2)}$,$\varvec{\upvarepsilon }^{(2)}$,$\varvec{\upsigma }^{(2)}$
bottom-of-the-crack (b) and top-of-the-crack (t) nodal displacement components	$u_\textrm{1b}$, $u_\textrm{2b}$, $u_\textrm{1t}$, $u_\textrm{2t}$
coordinates	$\textbf{x}$
coordinates mapping point in Equation 3	$\overline{\textbf{x}}$
dimensions of the rectangular domain of analysis	*w*, *h*
interaction strain energy	$W^{(1,2)}$
boundary of $\Omega$	$\Gamma$
contours in Equation 26	$\Gamma _\textrm{I}$,$\Gamma _\textrm{0}$,$\Gamma _\textrm{c}^+$,$\Gamma _\textrm{s}$, $\Gamma _\textrm{c}^-$
Kronecker delta	$\delta _{1j}$
virtual strains of Equation 5	$\Delta \varvec{\upvarepsilon }^\textrm{v}$
virtual displacements of Equation 5	$\Delta \textbf{u}^\textrm{v}$
numerical strain tensor	$\varvec{\upvarepsilon }$
exact strain tensor	$\varvec{\upvarepsilon }^\textrm{h}$
displacements relative errors	$\epsilon _1$, $\epsilon _2$
crack propagation angle	$\theta _\textrm{p}$
proportionality coefficients in Equation 22	$\lambda$,$\mu$
Poisson ratio	$\nu$
coordinates of Gauss points	$\xi$,$\eta$
von Mises stress	$\sigma _\textrm{vm}$
tip branch functions	$\psi _1^{k}(\textbf{x})$, $\psi _2^k(\textbf{x})$
angle of the crack with respect to horizontal	$\varphi$
domain of analysis	$\Omega$

## Basis of XFEM

In XFEM, additional nodes are introduced alongside standard nodes to form enrichment, thereby increasing the number of Degrees of Freedom (DOFs) within the whole domain^32,33^. When external forces are applied to the domain, these additional DOFs capture the discontinuity of nodal displacements on the two opposite sides of a crack.

**Fig. 1:**
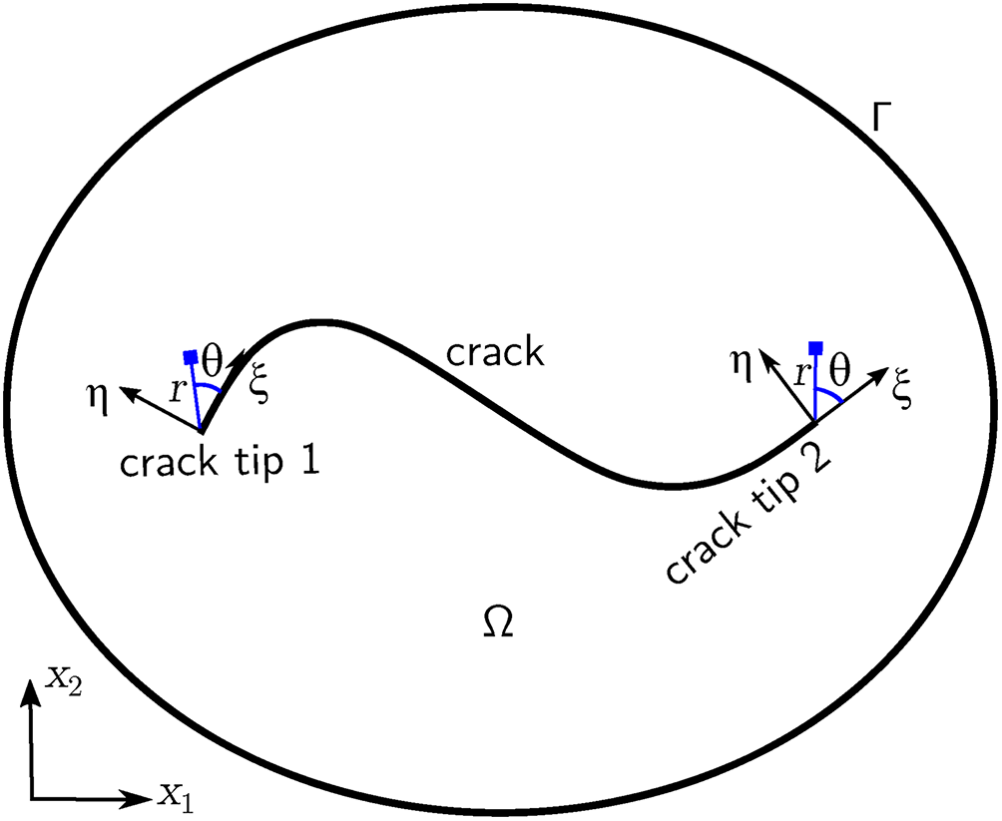
Schematic of a crack in domain.

With reference to Figure 1, for a single crack in a domain $\Omega$ bounded by $\Gamma$, by employing PUM, an approximation of the displacements $\textbf{u}(\textbf{x})$ can be obtained through the enriched shape functions with enriched DOFs^34^:

1 $$\begin{aligned} \begin{aligned} \textbf{u}(\textbf{x})=&\sum _{i=1}^{n} N_i(\textbf{x}) \textbf{u}_i+\sum _{j=1}^{n^{\textrm{H}}} N_j(\textbf{x})\left[ H(\textbf{x})-H\left( \textbf{x}_j\right) \right] \textbf{a}_j+\\&\sum _{k=1}^{n^{\textrm{tip1}}} N_k(\textbf{x})\left[ \psi _k^1(\textbf{x})-\psi _k^1\left( \textbf{x}_k\right) \right] \textbf{b}_k^1 +\sum _{k=1}^{n^{\textrm{tip2}}} N_k(\textbf{x})\left[ \psi _k^2(\textbf{x})-\psi _k^2\left( \textbf{x}_k\right) \right] \textbf{b}_k^2 \end{aligned} \end{aligned}$$

where *n* is the total number of standard nodes, $N(\textbf{x})$ are the standard shape functions, $\textbf{u}_i$ are the displacements of the standard nodes, $n^\textrm{H}$ is the number of Heaviside enriched nodes in the elements that the crack runs through, $H(\textbf{x})$ is the Heaviside enrichment function, $\textbf{a}_j$ are the displacements of the additional DOFs of the $n^\textrm{H}$ nodes, $n^\textrm{tip1}$ and $n^\textrm{tip2}$ are the numbers of tip enriched nodes near tip 1 and tip 2, respectively, $\psi ^1_k(\textbf{x})$ and $\psi ^2_k(\textbf{x})$ are the tip branch functions for crack tip 1 and crack tip 2, respectively, and $\textbf{b}_k^1$, $\textbf{b}_k^2$ are the additional DOFs of the nodes $n^\textrm{tip1}$ and $n^\textrm{tip2}$ respectively. $H(\textbf{x})$ serves as a jump function to represent the discontinuity across elements intersected by the crack, and it is expressed as^33^

2 $$\begin{aligned} H(\varphi (\textbf{x}))= {\left\{ \begin{array}{ll}1 & \varphi (\textbf{x}) \ge 0 \\ -1 & \varphi (\textbf{x})<0\end{array}\right. } \end{aligned}$$

where $\varphi (\textbf{x})$ is the level set function, written as

3 $$\begin{aligned} \varphi (\textbf{x})=\min \Vert \textbf{x}-\overline{\textbf{x}}\Vert {\text {sign}}(\textbf{n} \cdot (\textbf{x}-\overline{\textbf{x}})) \end{aligned}$$

being $\textbf{n}$ the unit vector normal to the crack, $\textbf{x}$ a generic point, and $\overline{\textbf{x}}$ the corresponding mapping point on the crack.

A general form of the tip branch function $\psi$ is as follows^35^:

4 $$\begin{aligned} \begin{aligned} \displaystyle \{\psi (r, \theta )\}_{i=1}^4&=\left[ \psi _i(\textbf{x}), i=1,2,3,4\right] \\&=\left[ \sqrt{r} \sin \frac{\theta }{2}, \sqrt{r} \sin \frac{\theta }{2} \sin \theta , \sqrt{r} \cos \frac{\theta }{2}, \sqrt{r} \cos \frac{\theta }{2} \sin \theta \right] \end{aligned} \end{aligned}$$

where *r* and $\theta$ are the polar coordinates of $\textbf{x}$ from the crack tips (1 or 2), $\theta$ measured counterclockwise from coordinate $\xi$, tangent to the crack at the tip (Figure 1). According to the principle of virtual work, assuming virtual displacements $\Delta \textbf{u}_\textrm{v}$ in $\Omega$, the following virtual work equation can be written:

5 $$\begin{aligned} \int _\Omega \varvec{\upsigma }: \Delta \varvec{\upvarepsilon }^\textrm{v} \textrm{d}\Omega =\int _\Omega \textbf{F}_\textrm{b} \cdot \Delta \textbf{u}^\textrm{v} \textrm{d}\Omega +\int _{\Gamma } \textbf{F}_\textrm{s} \cdot \Delta \textbf{u}^\textrm{v} \textrm{d}\Gamma +\textbf{F} \cdot \Delta \textbf{u}^\textrm{v} \end{aligned}$$

where $\varvec{\upsigma }$ is the stress tensor of the current state, $\Delta \varvec{\upvarepsilon }_\textrm{v}$ are the virtual strains resulting from $\Delta \textbf{u}_\textrm{v}$, and $\textbf{F}_\textrm{b}$, $\textbf{F}_\textrm{s}$, $\textbf{F}$ are body forces, surface forces on boundary $\Gamma$, and concentrated forces, respectively.

Given a FEM discretisation, the following equation can be written for an elastic material:

6 $$\begin{aligned} \textbf{K}\textbf{u}=\textbf{P} \end{aligned}$$

where $\textbf{K}$ is the global stiffness matrix, and $\textbf{P}$ is the load vector. The matrix $\textbf{K}$ can be calculated as

7 $$\begin{aligned} \textbf{K}=\int _{\Omega } \textbf{B}_i^T \textbf{D B}_j \textrm{d} \Omega \quad (i,j=\textrm{u,a,b}) \end{aligned}$$

where $\textbf{D}$ is the elasticity tensor, and $\textbf{B}_i$ are components of the strain differential operator matrix $\textbf{B}$, with u, a, and b referring to the standard nodes, the Heaviside enriched nodes, and the tip enriched nodes matrix, respectively.

For all the computations reported in what follows, Equation 6 and Equation 7 have been implemented into a dedicated code.

## Nodal displacements in a central-cracked domain

FEM and XFEM are computational methods relying on the quality and size of the mesh. The accuracy of the numerical simulation is crucial in modelling fracture growth and evolution as the trajectories are swayed even by slight errors throughout the iterations. In what follows, the sources of error are detected by analysing the deviation from the symmetry of the nodal displacements astride a crack, and expedients are suggested for enhancing the accuracy of the solution.

**Fig. 2:**
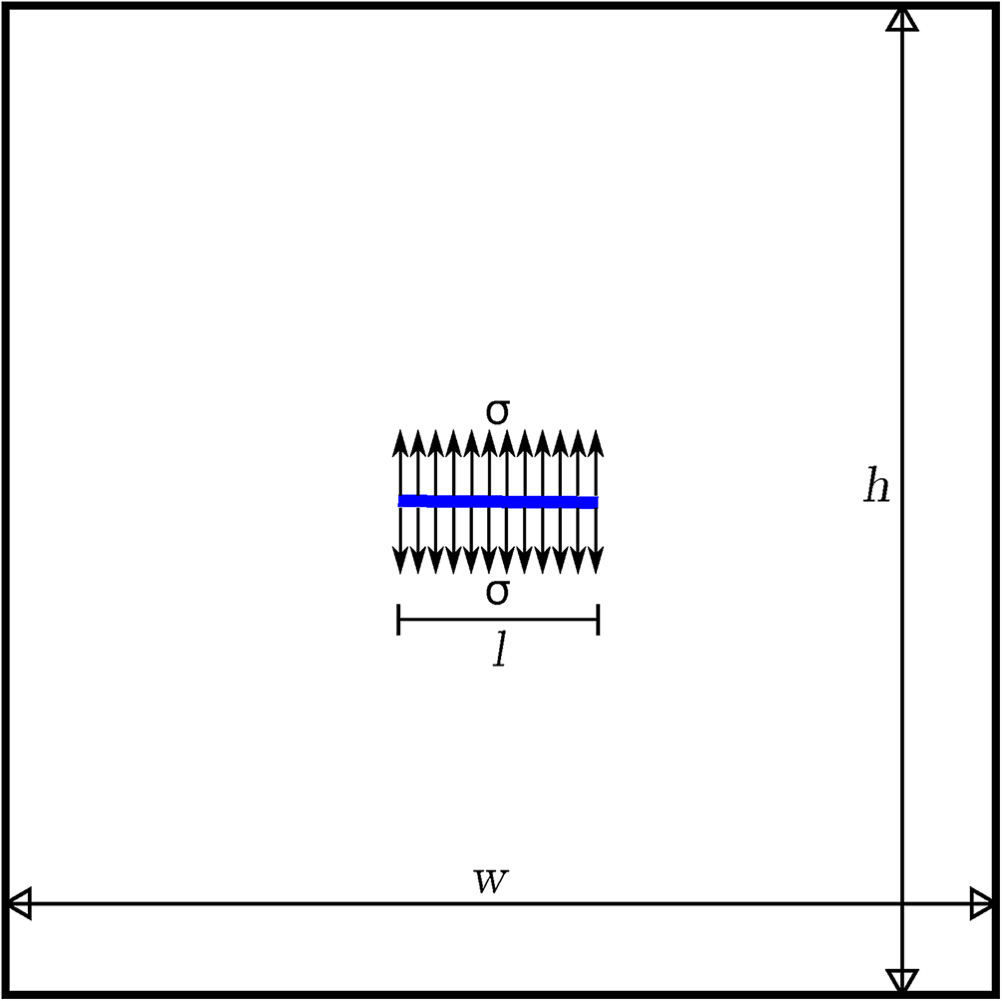
A $100 \times 100$ rectangular domain in plane strain with a central crack subjected to a pressure $\sigma$.

**Fig. 3:**
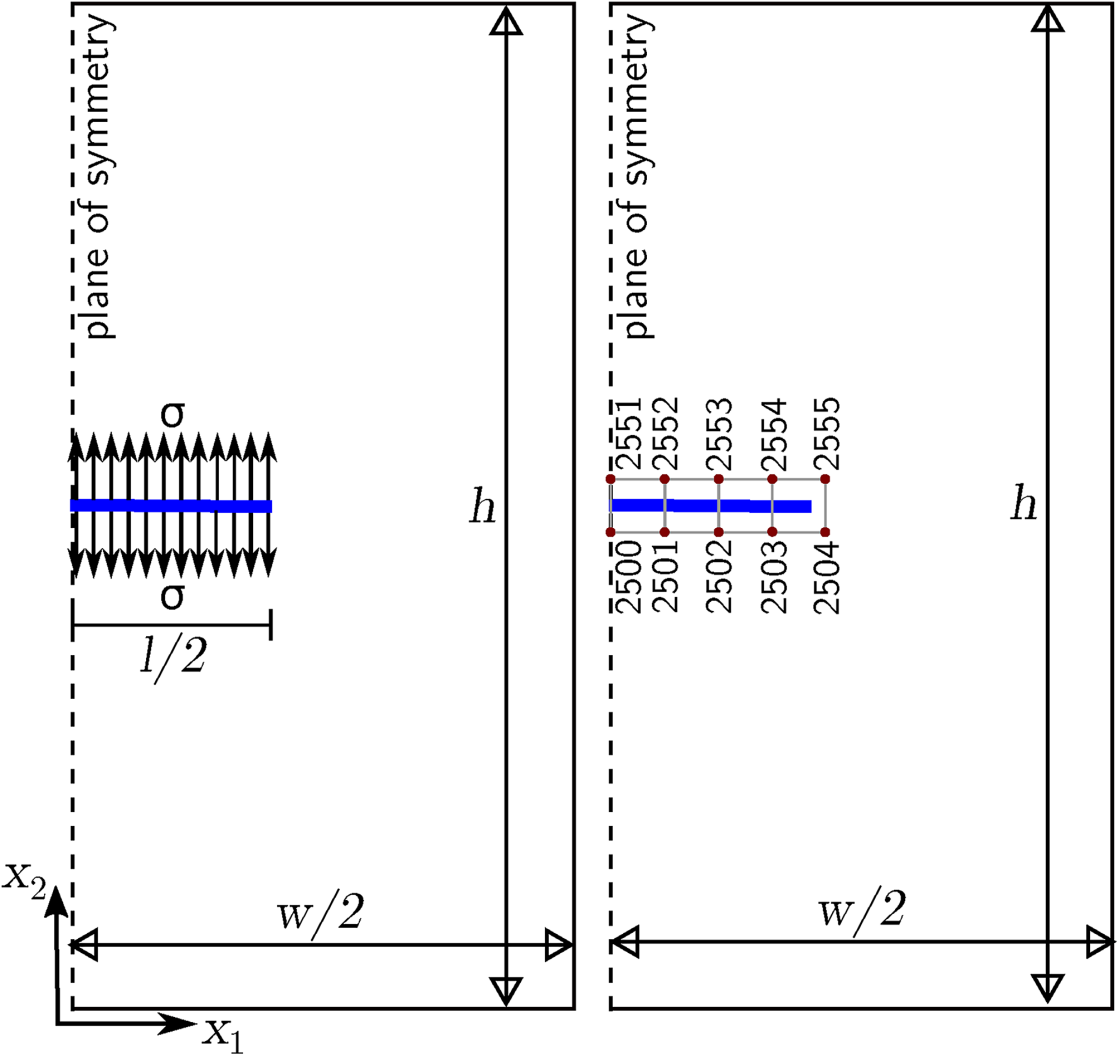
Half domain with edge crack ($50 \times 100$ mesh); (**a**) applied loads and boundary conditions; (**b**) nodal labels around the crack.

A plane strain model featuring a horizontal central crack is considered for this analysis (Figure 2). The domain is $$100\times 100\hbox {m}^{2}$$ square (*w*=*h*), with an elastic material having elastic modulus *E*$$5 \times 10$$$$^9$$Pa and Poisson ratio $$\nu$$ 0.25, and with a central crack of length *l* equal to 7m. A uniform pressure $$\sigma$$ of $$6 \times 10$$$$^6$$Pa is applied on both faces of the crack to promote opening. Given the symmetry, the domain is split into two subdomains by the vertical middle line (see Figure 3a). The boundary conditions on the subdomain are as follows: fixed horizontal displacement along the axis of symmetry, and restraint-free condition on the other sides. Four-node quadrilateral elements are used and three different meshes (with $$25 \times 50$$, $$50 \times 100$$, and $$100 \times 200$$ elements) are tested to assess the sensitivity of the solution to the element size.

**Table 2:** Displacements of the nodes astride the crack for different meshes.

	b/t nodes	$u_\textrm{1b}$(cm)	$u_\textrm{1t}$(cm)	$\epsilon _1$	$u_\textrm{2b}$(cm)	$u_\textrm{2t}$(cm)	$\epsilon _2$
$\textsf {25} \times \textsf {50}$	625/651	0	0	0%	−1.440139	1.423692	1.11%
	626/652	−0.266196	−0.257282	3.35%	−0.982162	0.971528	1.08%
	627/653	−0.364776	−0.352445	3.38%	−0.123090	0.134617	9.36%
$\textsf {50} \times \textsf {100}$	2500/2551	0	0	0%	−1.103186	1.101015	0.19%
	2501/2552	−0.079421	−0.079180	0.89%	−1.047912	1.045375	0.24%
	2502/2553	−0.135247	−0.134595	0.48%	−0.922175	0.917316	0.53%
	2503/2554	−0.224203	−0.218095	2.72%	−0.607094	0.600957	0.10%
$\textsf {50} \times \textsf {100}$	10000/10101	0	0	0	−0.966935	0.965817	0.11%
	10001/10102	−0.038211	−0.037893	0.83%	−0.929068	0.928049	0.10%
	10002/10103	−0.069104	−0.068524	0.83%	−0.946470	0.945439	0.10%
	10003/10104	−0.101041	−0.100201	0.83%	−0.934495	0.933439	0.11%
	10004/10105	−0.137217	−0.136095	0.81%	−0.904651	0.903479	0.12%
	10005/10106	−0.171298	−0.169814	0.86%	−0.859715	0.858090	0.18%
	10006/10107	−0.206202	−0.204962	0.60%	−0.746884	0.743322	0.47%
	10007/10108	−0.269666	−0.264127	2.00%	−0.480562	0.476074	0.93%

For the $50 \times 100$ mesh, the labels of the crack nodes astride the crack (the nodes of the elements crossed by the crack, ‘top’ (t) nodes above the crack, ‘bottom’ (b) nodes below the crack) are displayed in Figure 3b. Given the symmetry, for a pair of nodes with the same $x_1$ coordinate, $u_1$ of the b node ($u_\textrm{1b}$) must be equal to u1 of the t node ($u_\textrm{1t}$), and $u_2$ of the b node ($u_\textrm{2b}$) must be equal in absolute terms but opposite to $u_2$ of the t node ($u_{2t}$). The computed values of these nodal displacements $u_\textrm{1b}$, $u_\textrm{1t}$, $u_\textrm{2b}$, $u_\textrm{2t}$, are reported in Table 2, respectively for the meshes $25 \times 50$, $50 \times 100$, $100 \times 200$, together with the relative errors $\epsilon _1$, equal to ($|u_\textrm{1b}$-$u_\textrm{1t}|$)/$|u_\textrm{1b}|$, and $\epsilon _2$, equal to ($|u_\textrm{2b}$-$u_\textrm{2t}|$)/$|u_\textrm{2b}|$. It can be stated that the t and b displacements are not fully symmetrical and the errors $\epsilon _1$ and $\epsilon _2$ decrease with the mesh refinement, as expected.

## Analysis of asymmetry and improvements

As previously mentioned, the nodal displacements calculated by XFEM for pairing nodes astride the crack exhibit different offsets (see Table 2). This discrepancy (asymmetry) is attributed to the enrichment effect introduced by the additional nodes. To verify the assumption, in what follows the structure of the matrices $\textbf{B}_\textrm{u}$, $\textbf{B}_\textrm{a}$, and $\textbf{B}_\textrm{b}$ are analysed. Then, improvements are proposed to gain the symmetry, thereby enhancing the accuracy of the XFEM calculation.

For the analysis, the second simulation of the domain with central crack ($50 \times 100$), described in the above section, is considered.

In the first simulation, there are only standard nodes, thereby excluding any of the enrichments and with no computation of Heaviside and the tip branch function enrichments. It should be emphasised that this simulation is performed only for comparison. In Table 3, a comparison of the values of the nodal displacements $u_\textrm{1b}$, $u_\textrm{1t}$, $u_\textrm{2b}$, $u_\textrm{2t}$ of the nodes of Figure 3b is reported; the values, albeit strongly approximated, conform to the geometrical symmetry, thus the asymmetry of nodal displacements is caused by the enrichment functions.

**Table 3:** Displacements of the nodes astride the crack for the simulation without enrichment and only with Heaviside enrichment ($50 \times 100$ mesh).

	b/t nodes	$u_\textrm{1b}$(cm)	$u_\textrm{1t}$(cm)	$u_\textrm{2b}$(cm)	$u_\textrm{2t}$(cm)
without enr.	2500/2551	0	0	−0.061663905	0.061663905
	2501/2552	−3.13E-07	−3.13E-07	−0.070400638	0.070400638
	2502/2553	−0.019261999	−0.019261999	−0.089911865	0.089911865
	2503/2554	−0.058165116	−0.058165116	−0.092021259	0.092021259
with Heav.	2500/2551	0	0	−1.565229623	1.565229623
	2501/2552	−0.089770369	−0.089770369	−1.538961159	1.538961159
	2502/2553	−0.164682603	−0.164682603	−1.497103709	1.497103709
	2503/2554	−0.174688944	−0.174688944	−1.133059079	1.133059079

In the second simulation, Heaviside enriched nodes are introduced into the node set, requiring the reassembly of the global stiffness matrix $\textbf{K}$ to accommodate the expansion. The same calculation procedures as in the first simulation are followed, and the new values of the nodal displacements are also shown in Table 3. It can be seen that the upper nodes have the same displacements in absolute terms as the lower nodes, so the introduction of the Heaviside enrichment has no noticeable influence on inducing the numerical asymmetry.

In the third simulation, the crack tip enrichment is incorporated into the global framework. The displacements of the nodes of Figure 3b are those reported in Table 2 and are asymmetrical, therefore, the numerical asymmetry is mainly caused by the introduction of the tip enrichment functions.

The composition of the enrichment functions is analysed to identify the source of such errors. According to Equation 1 and Equation 7, the strain differential operator matrix $\textbf{B}_\textrm{a}$ for the Heaviside enrichment for node *i* can be expressed as:

8 $$\begin{aligned} \textbf{B}_\textrm{a}^i=\textbf{L}\bigl [ N_i(\textbf{x})\bigl (H(\textbf{x})-H(\textbf{x}_i)\bigr )\bigr ] \end{aligned}$$

with

9 $$\begin{aligned} \textbf{L}=\left[ \begin{array}{ccc} \frac{\partial }{\partial {x_1} } & 0 & \frac{\partial }{\partial {x_2} }\\ 0 & \frac{\partial }{\partial {x_2} } & \frac{\partial }{\partial {x_1} } \end{array}\right] ^\textrm{T} \end{aligned}$$

**Fig. 4:**
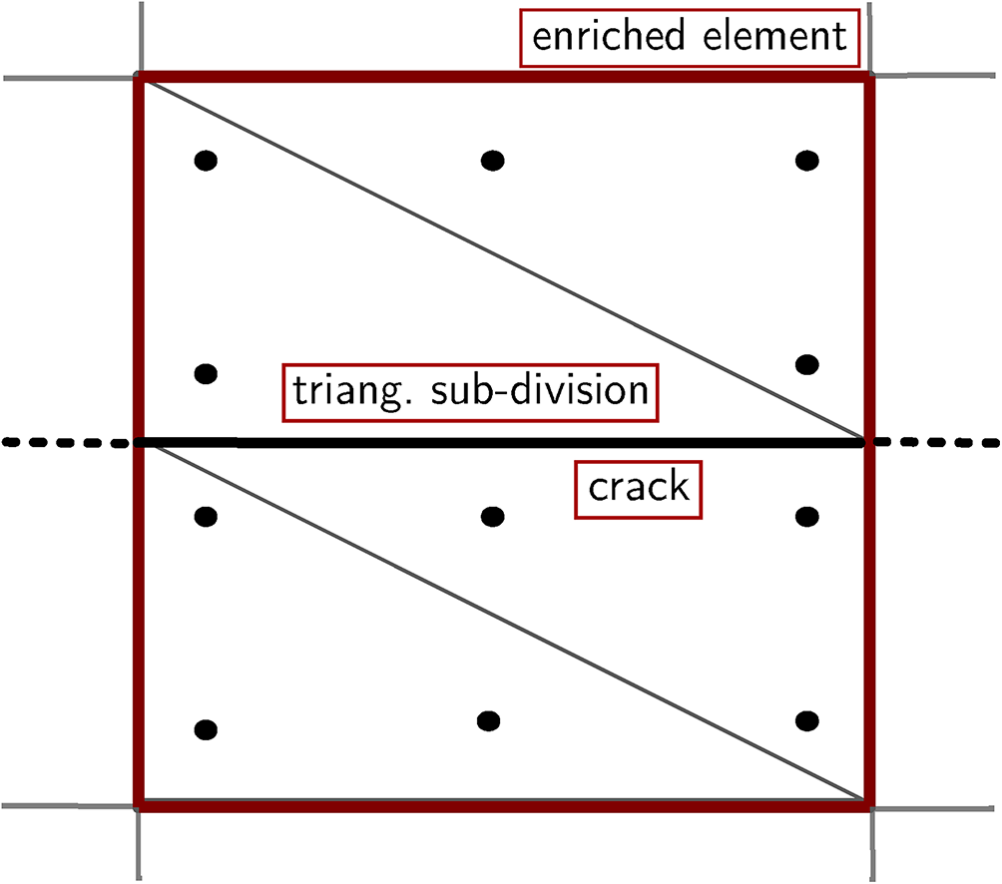
Distribution of Gauss points of Heaviside enriched element.

For the numerical computation of $\textbf{B}_\textrm{a}^i$, the distribution of Gauss points in Figure 4 is used. The contribution of a Gauss point G$_\textrm{p}$ is as follows:

10 $$\begin{aligned} \left[ \textbf{B}_\textrm{a}^i\right] _\textrm{Gp}=\Bigl [\textbf{L}\bigl [N_i(\textbf{x})\bigl (H(\textbf{x})- H(\textbf{x}_i)\bigr )\bigr ]\Bigl ]_\textrm{Gp} \end{aligned}$$

Since $H(\textbf{x}$) is a step function, the value is constrained to either 1 or -1, depending on the relative position of the Gauss point with respect to the crack. When the Gauss points are located on the same side of the crack as the corresponding node in Equation 10, the value of $H(\textbf{x})$-$H(\textbf{x}_i)$ is 0 whereas, if the Gauss point is on the positive axis and the node is on the negative axis, the value is 2; conversely, if the Gauss point is on the negative axis and the node is on the positive axis, the value is -2. Note that $H(\textbf{x})$-$H(\textbf{x}_i)$ remains constant. Consequently, Equation 10 can be transformed as follows:

11 $$\begin{aligned} {\left[ \textbf{B}_\textrm{a}^i\right] _\textrm{Gp} } =\bigl [\textbf{B}_\textrm{u}^i\bigl (H_i(\textbf{x})-H(\textbf{x}_i)\bigr )\bigr ]_\textrm{Gp} =A_\textrm{Gp}\left[ \textbf{B}_\textrm{u}^i\right] _\textrm{Gp} \end{aligned}$$

where $A_\textrm{Gp}$ is a constant, depending on the relative positions of the Gauss point and node with respect to the crack. According to Equation 11, for a given Gauss point, the value of $[\textbf{B}_\textrm{a}^i]_\textrm{Gp}$ is always a scalar multiple of the standard strain matrix $[\textbf{B}_\textrm{u}^i]_\textrm{Gp}$. As a result, the integrated element strain differential operator matrix $\textbf{B}_\textrm{a}$, computed through the combination of the values of all Gauss points, is also a scalar multiple of the standard strain matrix $\textbf{B}_\textrm{u}$.

Similarly, the strain differential operator matrix $\textbf{B}_\textrm{b}$ for the tip enrichments in Equation 7 can be expressed as:

12 $$\begin{aligned} \textbf{B}_\textrm{b}^i=\textbf{L}\bigl [ N_i(\textbf{x})\bigl (\Psi (\textbf{x})-\Psi (\textbf{x}_i)\bigr )\bigr ] \end{aligned}$$

With reference to Equation 4, it is known that both the shape function *N* and the tip enriched function $\psi$ are functions of the coordinates *x*, so, for a Gauss point, $\textbf{B}_\textrm{b}$ can be transformed as

13 $$\begin{aligned} \begin{aligned} \left[ \textbf{B}_\textrm{b}^i\right] _\textrm{Gp} =&\Bigl [ \textbf{L} \bigl [N_i(\textbf{x})\bigl (\Psi (\textbf{x})-\Psi (\textbf{x}_i)\bigr )\bigl ]\Bigr ]_\textrm{Gp} = \\&\left[ \textbf{L} N_i(\textbf{x})\right] _\textrm{Gp}\left[ \Psi (\textbf{x})-\Psi \left( \textbf{x}_i\right) \right] _\textrm{Gp} + \left[ N_i\right] _\textrm{Gp}\bigl [ \textbf{L} \bigl (\Psi (\textbf{x})-\Psi (\textbf{x}_i)\bigr )\bigr ]_\textrm{Gp} =\\&\left[ \textbf{B}_\textrm{u}^i\right] _\textrm{Gp}\left[ \Psi (\textbf{x})-\Psi \left( \textbf{x}_i\right) \right] _\textrm{Gp} + \left[ N_i\right] _\textrm{Gp}\bigl [ \textbf{L} \bigl (\Psi (\textbf{x})-\Psi (\textbf{x}_i)\bigr )\bigr ]_\textrm{Gp} \end{aligned} \end{aligned}$$

As a tip function includes four sub-functions, $\textbf{B}_\textrm{b}^i$ can be expressed in terms of four sub-matrices $\textbf{B}_\textrm{b}^i(1)$, $\textbf{B}_\textrm{b}^i(2)$, $\textbf{B}_\textrm{b}^i(3)$, and $\textbf{B}_\textrm{b}^i(4)$ as follows:

14 $$\begin{aligned} \begin{aligned} \textbf{B}_\textrm{b}^i(1)=&\begin{bmatrix} N_{i,1} c_1+N_i P_1(1) & 0\\ 0 & N_{i,2}c_1+N_iP_2(1) \\ N_{i,2} c_1+N_iP_2(1) & N_{i,1} c_1+N_i P_1(1) \end{bmatrix} \\ \textbf{B}_\textrm{b}^i(2)=&\begin{bmatrix} N_{i,1} c_2+N_iP_1(2) & 0\\ 0 & N_{i,2}c_2+N_iP_2(2)\\ N_{i,2}c_2+N_iP_2(2) & N_{i,1}c_2+N_iP_1(2) \end{bmatrix} \\ \textbf{B}_\textrm{b}^i(3)=&\begin{bmatrix} N_{i,1}c_3+N_iP_1(3) & 0\\ 0 & N_{i,2}c_3+N_iP_2(3)\\ N_{i,2}c_3+N_iP_2(3) & N_{i,1}c_3+N_iP_1(3) \end{bmatrix} \\ \textbf{B}_\textrm{b}^i(4)=&\begin{bmatrix} N_{i,1}c_4+N_iP_1(4) & 0\\ 0 & N_{i,2}c_4+N_iP_2(4)\\ N_{i,2}c_4+N_iP_2(4) & N_{i,1}c_4+N_iP_1(4) \end{bmatrix} \end{aligned} \end{aligned}$$

where $c_i$ are associated with the four branch equations in Equation 4 and $P_1(i)$ and $P_2(i)$ are as follows:

15 $$\begin{aligned} & {\left[ \begin{array}{l} P_1(1)\\ P_1(2)\\ P_1(3)\\ P_1(4) \end{array} \right] =\frac{1}{2 \sqrt{r}}\left[ \begin{array}{l} -\sin \frac{\theta }{2} \cos \varphi -\cos \frac{\theta }{2} \sin \varphi \\ \cos \frac{\theta }{2} \cos \varphi -\sin \frac{\theta }{2} \sin \varphi \\ -\sin \frac{3 \theta }{2} \sin \theta \cos \varphi -\left( \sin \frac{\theta }{2}+\sin \frac{3 \theta }{2} \cos \theta \right) \sin \varphi \\ -\cos \frac{3 \theta }{2} \sin \theta \cos \varphi -\left( \cos \frac{\theta }{2}+\cos \frac{3 \theta }{2} \cos \theta \right) \sin \varphi \end{array}\right] } \end{aligned}$$

16 $$\begin{aligned} & {\left[ \begin{array}{l} P_2(1)\\ P_2(2)\\ P_2(3)\\ P_2(4) \end{array} \right] = \frac{1}{2 \sqrt{r}}\left[ \begin{array}{l} -\sin \frac{\theta }{2} \sin \varphi +\cos \frac{\theta }{2} \cos \varphi \\ \cos \frac{\theta }{2} \sin \varphi +\sin \frac{\theta }{2} \cos \theta \\ -\sin \frac{3 \theta }{2} \sin \theta \sin \varphi +\left( \sin \frac{\theta }{2}+\sin \frac{3 \theta }{2} \cos \theta \right) \cos \varphi \\ -\cos \frac{3 \theta }{2} \sin \theta \sin \varphi +\left( \cos \frac{\theta }{2}+\cos \frac{3 \theta }{2} \cos \theta \right) \cos \varphi \end{array} \right] } \end{aligned}$$

where $\varphi$ is the crack angle with respect to horizontal. From Equation 13, Equation 14, Equation 15, and Equation 16, it is apparent that the derivation of the enriched shape functions includes an addition operation and an additional term, leading to a discrepancy between $\textbf{B}_\textrm{b}$ and $\textbf{B}_\textrm{u}$. It can be seen that $\textbf{B}_\textrm{u}$ comprising of $N_{i,1}$ and $N_{i,2}$ are symmetric, but adding extra terms ($N_iP_1$,$N_iP_2$) disables this symmetry as $P_1$ and $P_2$ are completely different for two symmetric Gauss points. Therefore, this discrepancy can be regarded as the underlying cause for the asymmetry observed in the solution for the displacements along the crack face.

### Improving the calculation of the nodal displacements

**Fig. 5:**
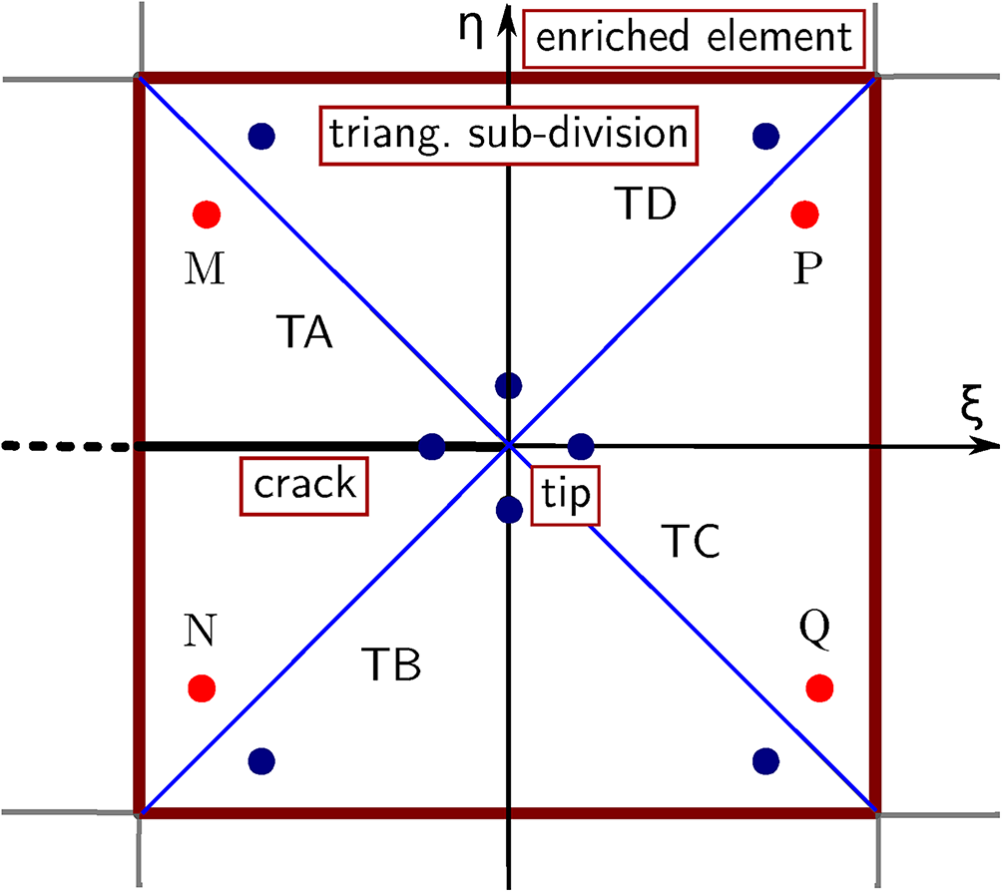
Schematic of T subdivisions around a crack tip (circles represent the Gauss points).

As shown in the previous section, the errors in calculating the displacements astride a crack can be reduced by refining the mesh. However, the operation often comes with significant computational costs and decreased efficiency. Therefore, a first expedient for the improvement of the solution is suggested: the quadratic elements containing the crack tip at the center are partitioned into equal triangular subdivisions as in Figure 5.

Three Gauss points are adopted for each triangular subdivision for the crack-tip enrichment functions, symmetrical with respect to the crack, resulting in symmetrical strain differential operator matrices. By exploiting this symmetry, the differences between the numerical displacement values of the symmetrical nodes are reduced.

As far as the tip-enriched elements are concerned, and with reference to the triangular subdivision TA in Figure 5, the contribution of the Gauss point M to the strain differential operator matrix $\textbf{B}_\textrm{b}$ is

17 $$\begin{aligned} \left[ \textbf{B}_\textrm{b}\right] _\textrm{M}=\textbf{B}_\textrm{b}(x_\textrm{M},y_\textrm{M}) \end{aligned}$$

Similarly, for Gauss point N, one has:

18 $$\begin{aligned} \left[ \textbf{B}_\textrm{b}\right] _\textrm{N}=\textbf{B}_\textrm{b}(x_\textrm{N},y_\textrm{N}) \end{aligned}$$

Since Gauss points M and N are symmetric with respect to the x-axis, one can conclude that:

19 $$\begin{aligned} \textbf{B}_\textrm{b}(x_\textrm{N},y_\textrm{N})=\textbf{B}_\textrm{b}(x_\textrm{M},-y_\textrm{M}) \end{aligned}$$

Similarly, for Gauss points P and Q in triangle TC, the contributions to Bb are:

20 $$\begin{aligned} \textbf{B}_\textrm{b}(x_\textrm{P},y_\textrm{P})=\textbf{B}_\textrm{b}(-x_\textrm{M},y_\textrm{M}) \end{aligned}$$

21 $$\begin{aligned} \textbf{B}_\textrm{b}(x_\textrm{Q},y_\textrm{Q})=\textbf{B}_\textrm{b}(-x_\textrm{M},-y_\textrm{M}) \end{aligned}$$

Based on the selected distribution of Gauss points, the same approach can be applied to process the rest of the Gauss points in the third quadrant. To assess the impact of this subdivision, the calculation results for the domain with central crack in Figure 3a are obtained using two different mesh configurations: $25 \times 50$ and $50 \times 100$, respectively. Equations 17 to 21 are substituted into Equation 13 to obtain $\textbf{B}_\textrm{b}$ which is in turn substituted in Equation 6 and Equation 7 to obtain the new nodal displacements of Table 4. By comparing the results with the solutions previously reported, the effectiveness of the proposed improvement is apparent.

**Table 4:** Displacements of the nodes astride the crack for the simulation with subdivisions in different meshes.

	b/t nodes	$u_\textrm{1b}$(cm)	$u_\textrm{1t}$(cm)	$\epsilon _1$	$u_\textrm{2b}$(cm)	$u_\textrm{2t}$(cm)	$\epsilon _2$
$\textsf{25} \times \textsf{50}$	625/651	0	0	0	−1.299808695	1.299808695	<0.01%
	626/652	−0.20842867	−0.20842867	<0.01%	−0.705972281	0.705972281	<0.01%
	627/653	−0.280216819	−0.280216819	<0.01%	0.011466637	0.011466637	<0.01%
$\textsf{50} \times \textsf{100}$	2500/2551	0	0	0	−1.026552948	1.026552917	<0.01%
	2501/2552	−0.086543716	−0.086543651	<0.01%	−0.964138038	0.964137989	<0.01%
	2502/2553	−0.148051385	−0.148051191	<0.01%	−0.838659958	0.838659949	<0.01%
	2503/2554	−0.193526767	−0.193526802	<0.01%	−0.444686605	0.444686632	<0.01%

However, when the crack-tip point is not positioned at the center of an element, the subdivisions after partitioning have different shapes and sizes, thus the Gauss points are no longer located symmetrically with respect to the crack and the expedient from Equation 17 to Equation 21 cannot be applied. One may resort to the expedient only when the coordinates of the Gauss points are identical, opposite, or proportional. The general format for two corresponding Gauss points is

22 $$\begin{aligned} \textbf{B}_\textrm{b}(x_\textrm{A},y_\textrm{A})=\textbf{B}_\textrm{b}(\lambda x_\textrm{M},\nu y_\textrm{M}) \end{aligned}$$

where $\lambda$ and $\mu$ are proportionality constants. A new code routine has been developed to identify Gauss points that possess the above characteristics. A second expedient to improve the solution is to increase the number of Gauss points assigned to each triangular subdivision. In this respect, it is useful to define an optimal number, therefore, in what follows, simulations of the domain with a central crack are performed by progressively increasing (7, 9, and 13) the number of Gauss points. The calculated displacements from the simulations are reported in Table 5, respectively.

**Table 5:** Displacements of the nodes astride the crack for the simulation with subdivisions and different numbers of Gauss points in each subdivision ($50 \times 100$ mesh).

	b/t nodes	$u_\textrm{1b}$(cm)	$u_\textrm{1t}$(cm)	$\epsilon _1$	$u_\textrm{2b}$(cm)	$u_\textrm{2t}$(cm)	$\epsilon _2$
7 Gauss p.	2500/2551	0	0	0	−1.103182172	1.100747027	0.22%
	2501/2552	−0.079746755	−0.079180418	0.71%	−1.047816074	1.045023662	0.26%
	2502/2553	−0.135731272	−0.135579677	0.11%	−0.921772297	0.918329593	0.37%
	2503/2554	−0.226823294	−0.219024456	3.34%	−0.606235018	0.600139992	1.00%
9 Gauss p.	2500/2551	0	0	0	−1.104810713	1.104597641	0.02%
	2501/2552	−0.079195404	−0.079345270	0.18%	−1.049607296	1.049299125	0.03%
	2502/2553	−0.134739447	−0.135225055	0.36%	−0.922963798	0.923387653	0.04%
	2503/2554	−0.223244723	−0.222162317	0.48%	−0.606416333	0.605154475	0.21%
13 Gauss p.	2500/2551	0	0	0	−1.09989233	1.099285495	0.05%
	2501/2552	-0.079135863	−0.078932963	0.25%	−1.044555398	1.043760466	0.07%
	2502/2553	−0.134435475	−0.134254384	0.13%	−0.918179068	0.916062984	0.23%
	2503/2554	−0.22133777	−0.219840794	0.67%	−0.598295641	0.595914318	0.39%

**Fig. 6:**
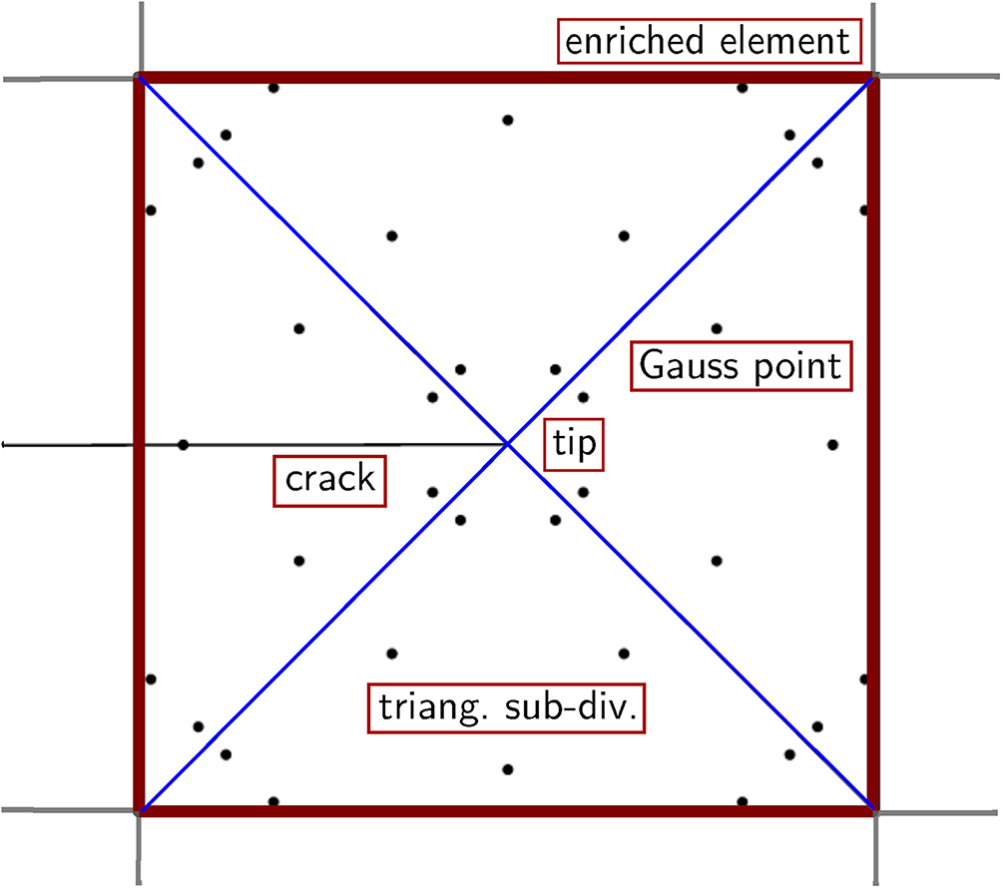
Locations of the 9 Gauss points in the triangular subdivisions around a crack tip.

By analysing the displacements of $50 \times 100$ mesh in Table 2 and Table 5, it can be seen that the displacements in Table  2 (3 Gauss points in each triangle) are affected by the largest error compared to the values reported in the other three tables. The errors decrease with increasing the number of Gauss points in a triangular subdivision. However, the error of the simulation with 13 Gauss points is only slightly greater than that of the simulation with 9 Gauss points, therefore, 9 Gauss points seem the optimal number and will be adopted in what follows. The locations of the 9 Gauss points in the subdivisions around a crack tip are shown in Figure 6.

To demonstrate the quality of the solution achieved by using subdivisions and 9 Gauss points in each subdivision, a simulation of the domain with a central crack is carried out and the results reported in Section 5 to estimate the SIF around the crack tip.

### Convergence study

**Fig. 7:**
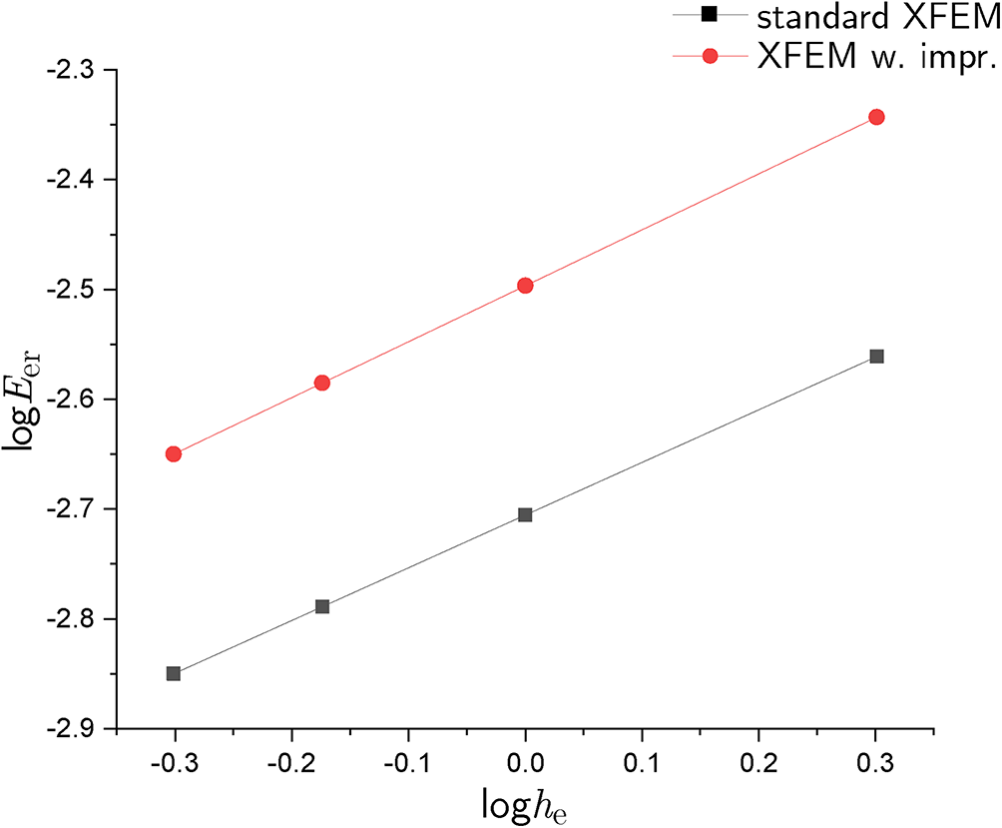
Convergence rates: red-line XFEM with improvements, black line standard XFEM.

To demonstrate the reliability of XFEM with the proposed improvements, a convergence study was conducted. In this section, four meshes are considered for a $50\times 100\hbox {m}^{2}$ domain: $25 \times 50$, $50 \times 100$, $75 \times 150$, $100 \times 200$. The element size $h_\textrm{e}$ reduces with the number of elements increasing. The convergence rate is associated with the variation of the relative error in energy norm $E_\textrm{er}$ with respect to $h_{e}$^36–38^. The formula for $E_\textrm{er}$ is:

23 $$\begin{aligned} E_\textrm{er}=\sqrt{\frac{1}{2}(\int _{\Omega } (\varvec{\upvarepsilon }-\varvec{\upvarepsilon }^\textrm{h})^{T}:\textbf{D}:(\varvec{\upvarepsilon }-\varvec{\upvarepsilon }^\textrm{h}) )} \end{aligned}$$

where $\varvec{\upvarepsilon }$ is the numerical strain tensor, $\varvec{\upvarepsilon }^\textrm{h}$ is the exact strain tensor and $\textbf{D}$ is the elasticity tensor. A comparison between the variation of $E_\textrm{er}$ obtained with standard XFEM and XFEM with improvements is shown in Figure 7, where $E_\textrm{er}$ is plotted against $h_\textrm{e}$ in logarithmic scale. The slope of each line is related to the corresponding convergence rate. The slopes for XFEM with improvements and standard XFEM are 0.51 and 0.48, respectively. The XFEM with improvements is therefore more convergent.

## SIF simulation example

### Model validation

In this section, the Interaction Integral Method (IIM)^39^ is employed to calculate the SIF Mode-I $K_\textrm{I}$ (opening mode) and SIF Mode-II $K_\textrm{II}$ (sliding mode) around the tip of the central crack of Figure 2. IIM is a highly accurate and effective method compared to other methods available for SIF calculations^40–42^.

**Fig. 8:**
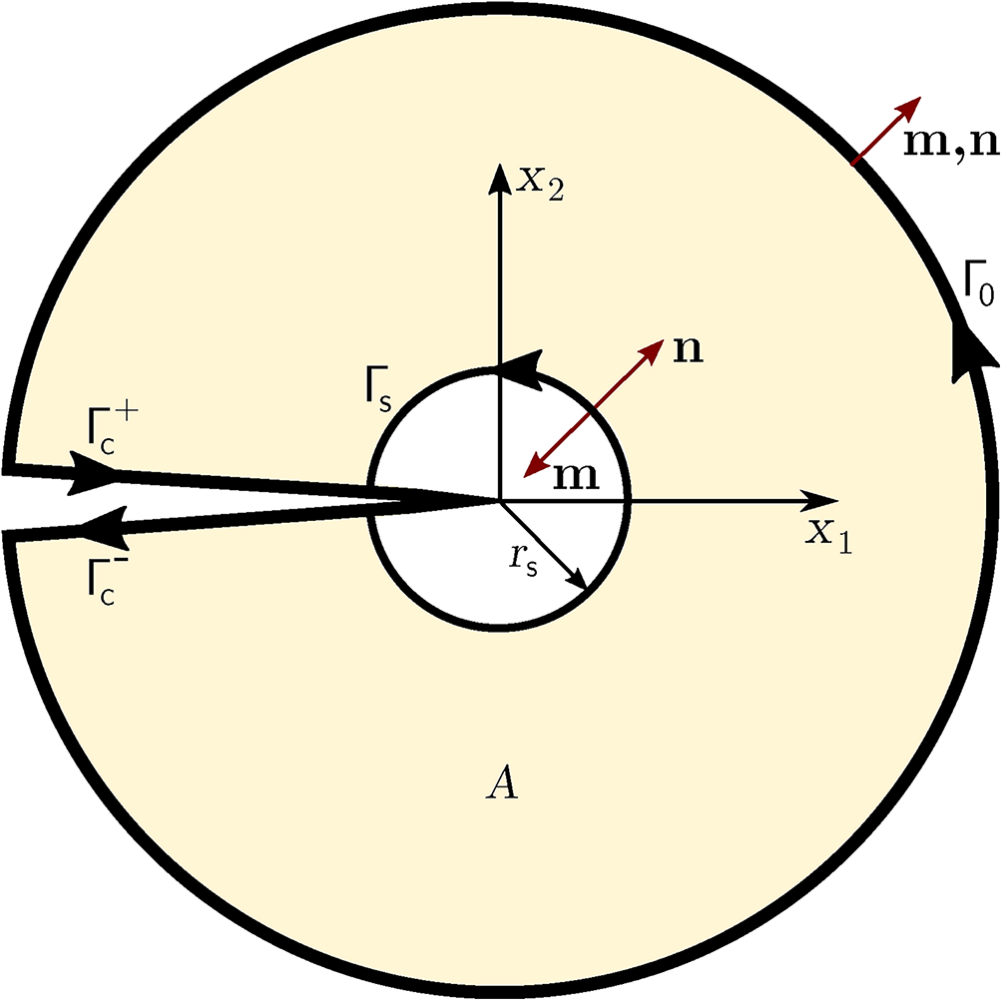
Schematic of the Interaction Integral Method (modified from^20^).

With reference to Figure 8, an inner circle centered in the crack tip and bounded by $\Gamma _0$ is considered. For a homogeneous material and a straight crack without surface stress, given current displacements $\textbf{u}^{(1)}$, strains $\varvec{\upvarepsilon }^{(1)}$, and stresses $\varvec{\upsigma }^{(1)}$, and corresponding auxiliary $\textbf{u}^{(2)}$, $\varvec{\upvarepsilon }^{(2)}$, $\varvec{\upsigma }^{(2)}$ functions (state (1) represents the current state, and state (2) represents the virtual state), the interaction integral $I^{(1,2)}$ can be expressed as^20^

24 $$\begin{aligned} I^{(1,2)}=\int _{\Gamma _\textrm{s}}\left[ W^{(1,2)}\delta _{1j}-\sigma ^{(1)}_{ij}\frac{\partial u_i^{(2)}}{\partial x_1}-\sigma ^{(2)}_{ij}\frac{\partial u_i^{(1)}}{\partial x_1}\right] n_j \textrm{d}\Gamma _\textrm{s} \end{aligned}$$

where $\Gamma _\textrm{s}$ is the boundary of a small circle of radius $r_\textrm{s}$ surrounding the crack tip, $\textbf{n}$ is the outward normal of $\Gamma _\textrm{s}$, $\delta _{1j}$ is the Kronecker delta, and $W^{(1,2)}$ is the interaction strain energy:

25 $$\begin{aligned} W^{(1,2)}=\sigma _{ij}^{(1)}\varepsilon _{ij}^{(2)}=\sigma _{ij}^{(2)}\varepsilon _{ij}^{(1)} \end{aligned}$$

The integral in Equation 24 is a boundary integral; for a finite element calculation, it can be transformed into an equivalent domain integral^28^. To this purpose, first, the integrand is multiplied by a weighting function $q(\textbf{x})$ equal to one and vanishing on $\Gamma _0$. Then for each $\Gamma _\textrm{s}$ Equation 24 is re-written as follows:

26 $$\begin{aligned} \begin{aligned} I^{(1,2)}=&\int _{\Gamma _\textrm{I}}\left[ \sigma _{i j}^{(1)} \frac{\partial u_i^{(2)}}{\partial x_1}+\sigma _{i j}^{(2)} \frac{\partial u_i^{(1)}}{\partial x_1}-W^{(1,2)} \delta _{1 j}\right] q m_j \textrm{d}\Gamma _\textrm{I}- \\&\int \limits _{\Gamma _\textrm{c}^+\cup \Gamma _\textrm{c}^-}\left[ \sigma _{i 2}^{(1)} \frac{\partial u_i^{(2)}}{\partial x_1}+\sigma _{i 2}^{(2)} \frac{\partial u_i^{(1)}}{\partial x_1}\right] q m_2 \mathrm {~d} \Gamma _\textrm{c} \end{aligned} \end{aligned}$$

where $\Gamma _\textrm{I}$=$\Gamma _0$+$\Gamma ^+_\textrm{c}$+$\Gamma _\textrm{s}$+$\Gamma _\textrm{c}^-$ and $\textbf{m}$ denotes a unit outward normal, equal to $\textbf{n}$ in $\Gamma _0$ and to -$\textbf{n}$ in $\Gamma _\textrm{s}$, and $m_1$=0 on $\Gamma _\textrm{c}^+$ and $\Gamma _\textrm{c}^-$.

**Fig. 9:**
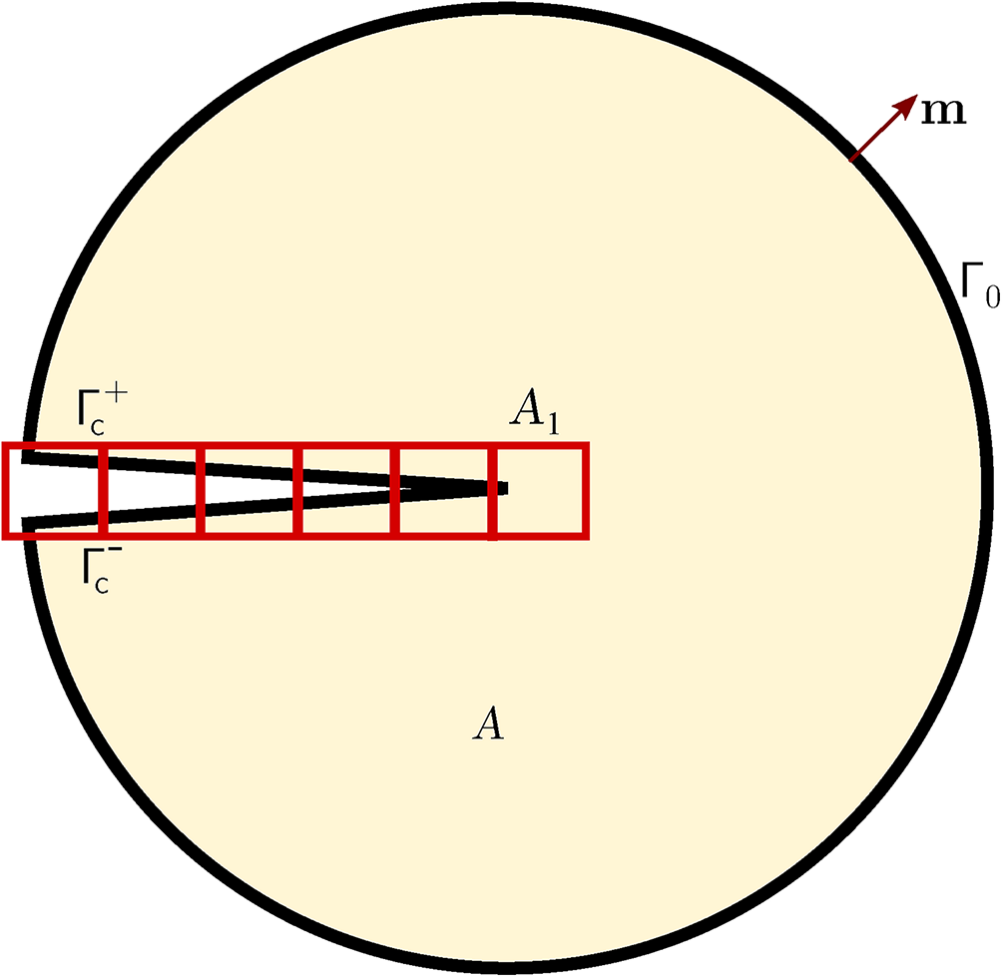
Enriched elements for the calculation of the interaction integral.

Given $\Gamma _{i2}m_2$ equal to 0 on the stress-free crack faces, by applying the divergence theorem and for $r_\textrm{s}\rightarrow$0, one has:

27 $$\begin{aligned} I^{(1,2)}=\int _A\left[ \sigma ^{(1)}_{ij}\frac{\partial u_i^{(2)}}{\partial x_1}+\sigma ^{(2)}_{ij}\frac{\partial u_i^{(1)}}{\partial x_1}-W^{(1,2)}\delta _{1j}\right] \frac{\partial q}{\partial x_j} \textrm{d}A \end{aligned}$$

where *A* is bounded like in Figure 8. However, in the case of tractions acting on the crack faces, the second integral in Equation 26 should be computed. To this purpose, a small domain composed of the connected elements enclosing the entire crack is considered. The contour $\Gamma _\textrm{c}^+$+$\Gamma _\textrm{c}^-$ is fictitiously extended to bound these elements, thus forming a distinct domain $A_1$ (Figure 9). Then, according to the divergence theorem, the contour integral can be transformed into a domain integral, and Equation 26 can be therefore approximated as follows:

28 $$\begin{aligned} \begin{aligned} I^{(1,2)}=&\int _A\left[ \sigma ^{(1)}_{ij}\frac{\partial u_i^{(2)}}{\partial x_1}+\sigma ^{(2)}_{ij}\frac{\partial u_i^{(1)}}{\partial x_1}-W^{(1,2)}\delta _{1j}\right] \frac{\partial q}{\partial x_j} \textrm{d}A- \\&\int _{A_1}\left[ \sigma ^{(1)}_{i2}\frac{\partial u_i^{(2)}}{\partial x_1}+\sigma ^{(2)}_{i2}\frac{\partial u_i^{(1)}}{\partial x_1}\right] \frac{\partial q}{\partial x_2} \textrm{d}A_1 \end{aligned} \end{aligned}$$

This expedient introduces a presumably small error, however, the overall computation result should not be affected much. Equation 28 can be specialised for the Heaviside-enriched elements as follows:

29 $$\begin{aligned} \begin{aligned} I^\textrm{H}=&\sum \limits _{n^\textrm{H}_\textrm{e}}\int _{\textrm{e}^\textrm{H}}\left[ \sigma ^{(1)}_{ij}\frac{\partial u_i^{(2)}}{\partial x_1}+\sigma ^{(2)}_{ij}\frac{\partial u_i^{(1)}}{\partial x_1}-W^{(1,2)}\delta _{1j}\right] \frac{\partial q}{\partial x_j} \textrm{d}\textrm{e}^\textrm{H}- \\&\sum \limits _{n^\textrm{H}_\textrm{e}}\int _{\textrm{e}^\textrm{H}}\left[ \sigma ^{(1)}_{i2}\frac{\partial u_i^{(2)}}{\partial x_1}+\sigma ^{(2)}_{i2}\frac{\partial u_i^{(1)}}{\partial x_1}\right] \frac{\partial q}{\partial x_2} \textrm{d}\textrm{e}^\textrm{H} \end{aligned} \end{aligned}$$

where $I^\textrm{H}$ is the remainder of the full integral within all the Heaviside-enriched elements subtracting the influence of face traction (H stands for Heaviside), $n^\textrm{H}_\textrm{e}$ is the number of Heaviside elements, and e$^\textrm{H}$ is a Heaviside-enriched element.

Similarly, for the tip-enriched elements one has:

30 $$\begin{aligned} \begin{aligned} I^\textrm{tip}=&\sum \limits _{n^\textrm{tip}_\textrm{e}}\int _{\textrm{e}^\textrm{tip}}\left[ \sigma ^{(1)}_{ij}\frac{\partial u_i^{(2)}}{\partial x_1}+\sigma ^{(2)}_{ij}\frac{\partial u_i^{(1)}}{\partial x_1}-W^{(1,2)}\delta _{1j}\right] \frac{\partial q}{\partial x_j} \textrm{d}\textrm{e}^\textrm{tip}- \\&\sum \limits _{n^\textrm{tip}_\textrm{e}}\int _{\textrm{e}^\textrm{tip}}\left[ \sigma ^{(1)}_{i2}\frac{\partial u_i^{(2)}}{\partial x_1}+\sigma ^{(2)}_{i2}\frac{\partial u_i^{(1)}}{\partial x_1}\right] \frac{\partial q}{\partial x_2} \textrm{d}\textrm{e}^\textrm{tip} \end{aligned} \end{aligned}$$

where $I^\textrm{tip}$ is the remainder of the full integral within the tip elements subtracting the influence of face traction (T stands for tip), $n^\textrm{tip}_\textrm{e}$ is the number of crack tip elements and e$^\textrm{tip}$ is a tip-enriched element at one extremity of the crack.

According to the above analysis, the whole interaction integral surrounding the crack tip equals the sum of $I^\textrm{H}$, $I^\textrm{tip}$, and the integral of elements outside $A_1$ but within *A*.

With reference to Figure 2, and for crack lengths equal to 7, 11, 15, and 19 metres, the numerical values of SIF obtained by using Equation 28, Equation 29, and Equation 30 are compared to the analytical solution^8^, expressed as:

31 $$\begin{aligned} K_\textrm{I}=\sigma \sqrt{\pi (l/2)} \end{aligned}$$

The results are reported in Table 6, alongside the results obtained with the standard XFEM solution. The XFEM solution adheres much more to the analytical solution thanks to the improvements.

**Table 6:** SIF $K_\textrm{I}$ values (in 10$^7$Pa/$\hbox {m}^2$) obtained with the analytical solution, the XFEM solution with improvements and the standard XFEM solution.

crack length(m)	7	11	15	19	Average error
Analytical	1.9890	2.4934	2.9117	3.2770	
XFEM with improvements	1.9811	2.4838	2.9558	3.2038	1.13%
Standard XFEM	1.9621	2.4953	2.9614	3.3977	2.94%

### Crack propagation

XFEM has advantages in studying crack propagation due to avoiding constant remeshing and element refinement. Near the crack tip, a highly concentrated stress field arises driving to crack growth, quantified by the SIF. For the simulation of the propagation, the effective SIF $K_\textrm{eff}$ is considered^43^:

32 $$\begin{aligned} K_\textrm{eff}=(K_\textrm{I}^4+8K_\textrm{II}^4)^{1/4} \end{aligned}$$

When $K_\textrm{eff}$ exceeds the crack toughness $K_\textrm{IC}$, the crack propagates along the direction of maximum circumferential stress; the corresponding angle of propagation $\theta _\textrm{p}$ measured like $\theta$ in Figure 1 is:

33 $$\begin{aligned} \theta _\textrm{p}=2\arctan \!\!\left[ \frac{1}{4}\left( \frac{K_\textrm{I}}{K_\textrm{II}}\pm \sqrt{\left( \frac{K_\textrm{I}}{K_\textrm{II}}\right) ^2+8}\right) \right] \end{aligned}$$

As previously mentioned, for the calculation of the SIF values, the Interaction Integral Method (IIM) is adopted herein.

**Fig. 10:**
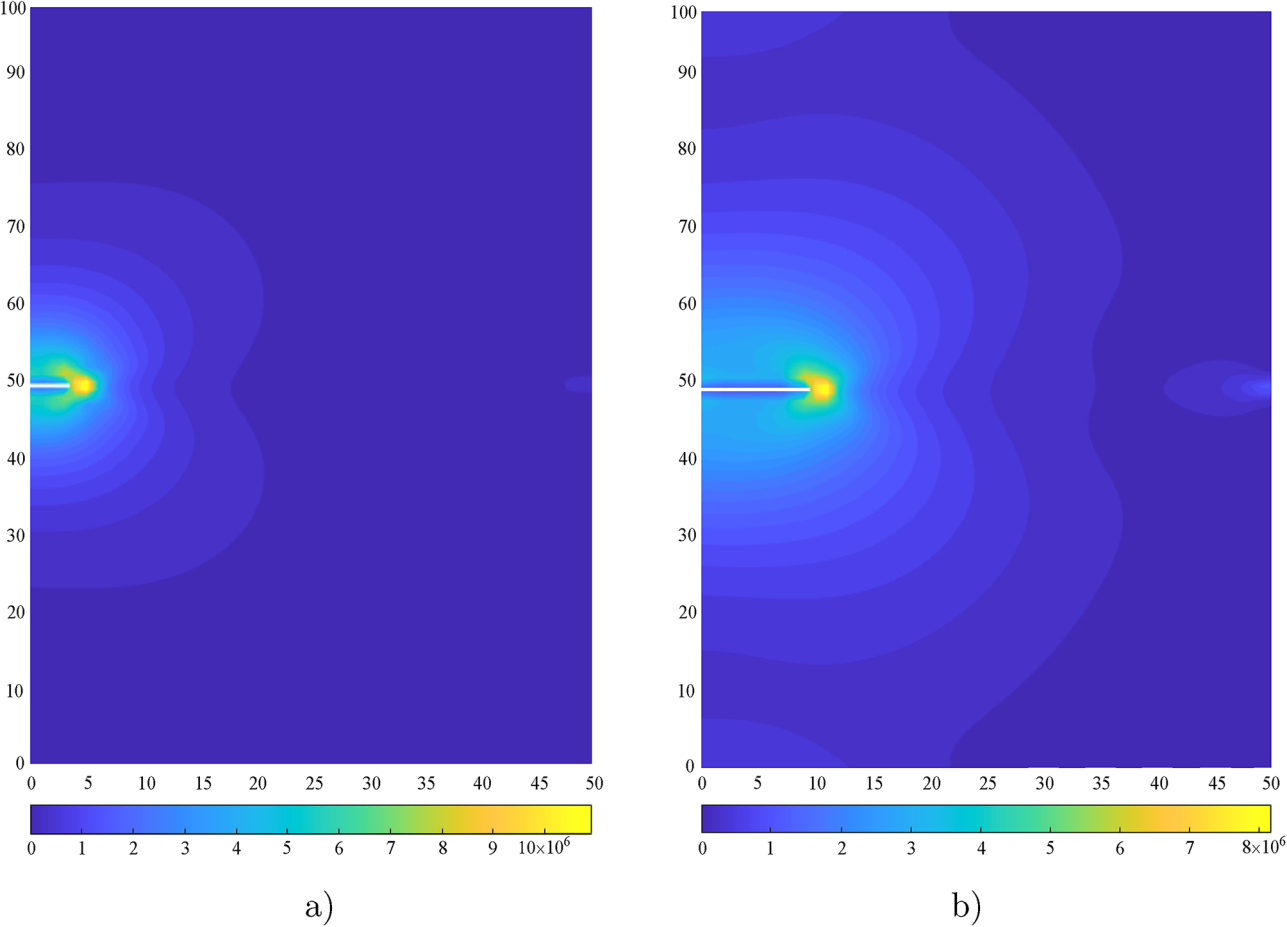
Distribution of von Mises stress $\sigma _\textrm{vm}$, (**a**) when crack is at the initial stage, (**b**) when propagated.

For the problem of Figure 2 (solution domain in Figure 3a), the distribution of the von Mises stress $\sigma _\textrm{vm}$ when the crack is at the initial length of 3.5m and when propagated is shown in Figure 10a and b, respectively. The results offered by the XFEM solution with improvements and those by the standard XFEM solution are reported in Table 7 in terms of nodal displacements astride the crack when the crack is propagated. It is apparent that, with reference to the difference between the displacements of top and bottom nodes astride the crack the application of the improvements leads to a more contained error. It is also found that by using XFEM with improvements no alteration of the crack path is experienced if compared to the one from standard XFEM. This is because both the solutions yield very similar SIF values, so, consequently, the corresponding simulated crack paths are nearly identical. The differences between the two solutions are primarily reflected in the displacements.

**Table 7:** Displacements astride the propagated crack for the XFEM solution with improvements and the standard XFEM solution.

	b/t nodes	$u_\textrm{1b}$(cm)	$u_\textrm{1t}$(cm)	$\epsilon _1$	$u_\textrm{2b}$(cm)	$u_\textrm{2t}$(cm)	$\epsilon _2$
XFEM imp.	2500/2551	0	0	0	−1.504538482	1.503047256	0.09%
	2501/2552	-0.058907427	−0.078932963	0.5%	−1.476208896	1.474828399	0.09%
	2502/2553	−0.105776559	−0.105141322	0.6%	−1.454521670	1.453138220	0.09%
	2503/2554	−0.152293273	−0.151361521	0.6%	−1.417814709	1.416424039	0.09%
stan. XFEM	2500/2551	0	0	0	−1.495380428	1.486683605	0.5%
	2501/2552	-0.060778525	−0.078932963	8%	−1.460739176	1.464871400	0.2%
	2502/2553	−0.105991238	−0.103419389	2%	−1.438885457	1.442970411	0.3%
	2503/2554	−0.150806453	−0.150470139	0.2%	−1.401898303	1.405863147	0.2%

### Discussion

Although XFEM provides significant advantages in modelling crack propagation and evolution, it is also subjected to several limitations and challenges. For instance, a precise definition of the initial crack position and geometry is required; any inaccuracy in this setup may compromise the quality of the results. Tracking crack paths become particularly challenging when cracks branch, merge, or intersect with others. Simulating multiple cracks and mutual interactions requires sophisticated algorithms to manage propagation and evolution. Consequently, careful selection and implementation of the enrichment functions near crack tips are necessary to minimize numerical errors. Despite these limitations, XFEM remains a robust tool for addressing problems of fracture mechanics, specifically those involving discontinuous stress fields and inclusion issues. In future research, the authors intend to extend the work to three-dimensional applications, aiming to expand XFEM use to a broader range of scenarios and demonstrate the feasibility of the method with the improvements in interdisciplinary contexts.

## Conclusion

In this paper, two improvements to the XFEM calculation are proposed to reduce the discrepancies among the nodal displacements astride a crack that theoretically should be symmetrical. The source of error behind such discrepancies is first investigated by analysing the structure of the stiffness matrix. It is found that the discrepancies are primarily caused by the derivation of the tip branch functions. It is shown that a mesh refinement can enhance the accuracy, however, it often comes with a cost. Therefore, it is alternatively proposed herein to 1) efficiently use the Gauss points by resorting to subdivisions of the enriched elements around the crack, provided that such points have the same, opposite, or proportional coordinates, and 2) define the optimal number of such Gauss points.

To demonstrate the effectiveness of such improvements, the numerical calculation of the SIF at the tip of cracks subjected to surface stress is compared to the analytical solution. The numerical calculation is performed via the application of the interaction integral technique, transforming the linear integral along the crack face into an area integral of elements comparison of the SIF values with the analytical solutions is encouraging.

## Data Availability

All data generated or analysed during this study are included in this published article.
